# Environmental Enrichment Protects the Retina from Early Diabetic Damage in Adult Rats

**DOI:** 10.1371/journal.pone.0101829

**Published:** 2014-07-08

**Authors:** Damián Dorfman, Marcos L. Aranda, María Florencia González Fleitas, Mónica S. Chianelli, Diego C. Fernandez, Pablo H. Sande, Ruth E. Rosenstein

**Affiliations:** 1 Laboratory of Retinal Neurochemistry and Experimental Ophthalmology, Department of Human Biochemistry, School of Medicine/CEFyBO, University of Buenos Aires/CONICET, Buenos Aires, Argentina; 2 Laboratory of Histology, School of Medicine, University of Morón, Buenos Aires, Argentina; Dalhousie University, Canada

## Abstract

Diabetic retinopathy is a leading cause of reduced visual acuity and acquired blindness. Available treatments are not completely effective. We analyzed the effect of environmental enrichment on retinal damage induced by experimental diabetes in adult *Wistar* rats. Diabetes was induced by an intraperitoneal injection of streptozotocin. Three days after vehicle or streptozotocin injection, animals were housed in enriched environment or remained in a standard environment. Retinal function (electroretinogram, and oscillatory potentials), retinal morphology, blood-retinal barrier integrity, synaptophysin, astrocyte and Müller cell glial fibrillary acidic protein, vascular endothelial growth factor, tumor necrosis factor-α, and brain-derived neurotrophic factor levels, as well as lipid peroxidation were assessed in retina from diabetic animals housed in standard or enriched environment. Environmental enrichment preserved scotopic electroretinogram a-wave, b-wave and oscillatory potential amplitude, avoided albumin-Evan's blue leakage, prevented the decrease in retinal synaptophysin and astrocyte glial fibrillary acidic protein levels, the increase in Müller cell glial fibrillary acidic protein, vascular endothelial growth factor and tumor necrosis factor-α levels, as well as oxidative stress induced by diabetes. In addition, enriched environment prevented the decrease in retinal brain-derived neurotrophic factor levels induced by experimental diabetes. When environmental enrichment started 7 weeks after diabetes onset, retinal function was significantly preserved. These results indicate that enriched environment could attenuate the early diabetic damage in the retina from adult rats.

## Introduction

Diabetic retinopathy (DR), a leading cause of reduced visual acuity and acquired blindness, is a retinal hyperglycemia-related ischemic disorder characterized by microvascular and neuroglial alterations [1]. Nearly all individuals with type 1 diabetes, and more than 60% of individuals with type 2 diabetes mellitus have some degree of retinopathy after 20 years [2]. Current population-based studies suggest that about one-third of the diabetic population have signs of DR, and approximately one tenth have vision-threatening stages of retinopathy [3], [4].

Like other diabetic complications, the exact mechanism underlying DR is still not completely understood. However, it is well recognized that DR is a disorder that leads to increased vascular permeability, neovascularization, edema, and lesions in the retina (reviewed in [5]). At present, the treatments for advanced stages of DR are laser photocoagulation, vitrectomy, and intraocular administration of anti- vascular endothelial growth factor (VEGF) agents, and corticosteroids. Although these treatments have had considerable success rates, they are not useful for early stages of DR, and do not completely eliminate the risk of blindness (reviewed in [6]). Thus, new treatment strategies that are preventive and/or can provide interventions in diabetes to delay or prevent the progression of the retinal disease are needed. The streptozotocin (STZ)-induced diabetes model in rat shows many of the retinal alterations associated with human DR [7], [8]; therefore, this model could be a useful tool for developing new therapeutic strategies for DR.

Environmental conditions have been shown to rescue the diabetic brain from neurodegenerative progression [9], and to prevent or delay the development of memory deficits caused by diabetes in adult rats [10]. In an enriched environment (EE), animals are allowed the freedom to move and exercise voluntarily in larger cages, with accessibility to complex stimuli (e.g. toys, running wheels, etc.), thus being provided with more sensorial, social, physical, and intellectual stimulation than animals housed in standard (laboratory) conditions. Compelling evidence exists for the influence of EE housing on enhanced neuronal growth, and recovery following central nervous system injuries [11]–[13]. Landi et al. [14] have shown that EE housing accelerates the maturation of rat retinal acuity and an enhanced visual acuity was demonstrated in mice exposed to EE from birth, which suggests that the complexity of the visual environment greatly influences the visual system performance [15]. Furthermore, remarkable therapeutic effects on the visual system after prolonged exposure to EE from birth of rd10 mice, a mutant strain undergoing progressive photoreceptor degeneration mimicking human retinitis pigmentosa, were recently demonstrated [16]. Although the adult retina has long been considered less plastic than the brain cortex or hippocampus (the main loci of experience-dependent plasticity), a marked upregulation of the nerve growth factor induced gene-A and the activity-regulated cytoskeletal protein, two candidate-plasticity genes, was described in adult rats that had been exposed to an EE for 3 weeks [17]. Moreover, we have recently demonstrated that EE housing after retinal ischemia significantly protects retinal function and histology from ischemia/reperfusion injury in adult rats [18], and a recent study has confirmed that the outcome of retinal ischemia is improved by enriched housing in adult rats [19], supporting that environmental stimuli can significantly modify the extent of retinal damage in adult rats. In this context, the aim of this work was to analyze the effect of EE housing on retinal damage induced by early experimental diabetes.

## Materials and Methods

### Ethics Statement

All animal procedures were in strict accordance with the ARVO Statement for the Use of Animals in Ophthalmic and Vision Research. The ethic committee of the School of Medicine, University of Buenos Aires (Institutional Committee for the Care and Use of Laboratory Animals, (CICUAL)) approved this study, and all efforts were made to minimize animal suffering.

### Animals

**Figure 1:**
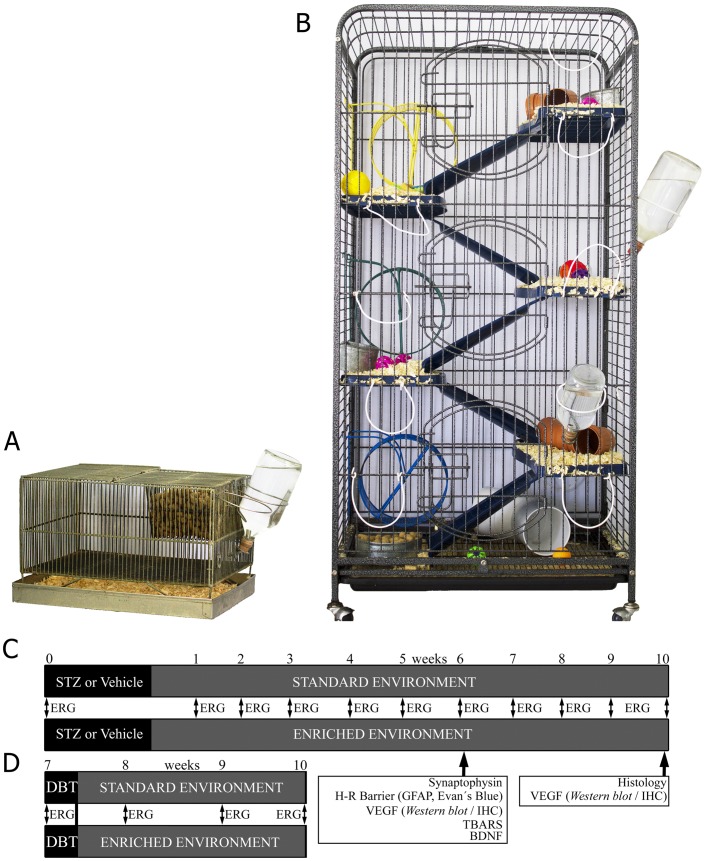
Housing conditions, and protocols for retinal functional, histological and biochemical studies. Three days after vehicle or STZ injection, adult *Wistar* rats were housed in a standard laboratory cage (panel A) or EE cages (panel B). Panel C: Experimental groups and protocols for functional, histological and biochemical studies. ERG was weekly recorded until week 10. Vascular permeability, GFAP immunostaining, retinal TNFα and TBARS levels were analyzed in SE- or EE-housed animals that had been diabetic for 6 weeks. VEGF levels were analyzed by Western blot and immunohistochemistry at 6 or 10 weeks of diabetes. Panel D: Diabetic animals housed in SE until week 7 were switched to EE, and ERG was weekly recorded until week 10.

Adult male *Wistar* rats (average weight, 300±50 g) were housed in a standard animal room with food and water *ad libitum*, under controlled conditions of humidity and temperature (21±2°C). The room was lighted by fluorescent lights (200 lux) that were turned on and off automatically every 12 hours (on from 8.00 AM to 8.00 PM). Animals from the control group (standard environment, (SE)) were housed in standard laboratory cages (33.5×45×21.5 cm) with two animals per cage. For EE housing, six animals at a time were housed in big metallic cages (46.5×78×95 cm), containing four floors and several food hoppers, water bottles, running wheels, tubes, ramps and differently shaped objects (balls, ropes, stones) repositioned once a day and fully substituted once a week. Particular care was taken not to repeat cage arrangement and object availability during the experiments, as previously described [18]. Animals were caged in EE at 3 days after STZ injection. Although food and water were offered *ad libitum*, location of the hoppers and bottles was changed daily in order to stimulate exploratory conduct. Cages were cleaned twice a week at the same time and by the same protocol to that used for standard cage cleaning [18]. For diabetes induction, a single intraperitoneal injection of streptozotocin (STZ, 60 mg/kg in 0.1 M citrate buffer, pH 4.5) was performed, whereas control rats received an equal volume of citrate buffer. Animals were examined 3 days after injections with a glucose meter (Bayer, Buenos Aires, Argentina), and those with glycaemia greater than 350 mg/dl were considered diabetic. Rats were weekly monitored and the weight and plasma glucose levels were determined. In another set of experiments, diabetic rats were housed in EE at 7 weeks of diabetes. The housing conditions for experimental groups, and protocols for retinal functional, histological and biochemical studies are shown in Figure 1. A total number of 185 animals were used for the experiments, distributed as follows: for ERG assessment during 10 weeks of diabetes: 22 control (10 in SE and 12 in EE) and 22 diabetic animals (10 in SE and 12 in EE). Five animals from each group were used for histological analysis and 5 animals were used for VEGF levels at 10 weeks of diabetes. For GFAP and Evan's Blue analysis: 13 animals/group were used. In addition, 20 animals were used to asses VEGF levels at 6 weeks of diabetes (5 SE-housed control or diabetic animals and 5 EE-housed control or diabetic animals. For lipid peroxidation assessment, 20 animals were used (5 control animals in SE or EE, and 5 diabetic animals in SE or EE). For immunohistochemical studies, 20 animals were used (5 control animals in SE or EE, and 5 diabetic animals in SE or EE). Finally, 32 animals were used to evaluate the effect of EE on the ERG from 7 to 10 weeks of diabetes (10 control animals in SE, 10 diabetic animals that remained in SE the whole experiment, and 12 animals that were changed from SE to EE at 7 weeks of diabetes).

### Cataract prevalence

Cataract (lens opalescence) development was weekly monitored using a handheld ophthalmoscope equipped with a slit lamp.

### Electroretinography

Electroretinograms (ERG) were recorded before and weekly after diabetes induction, as previously described [18], [20]. Briefly, rats were dark-adapted for 6 h, and anesthetized with ketamine hydrochloride (150 mg/kg) and xylazine hydrochloride (2 mg/kg) administered intraperitoneally, under dim red illumination. Pupils were dilated by topical application of phenylephrine hydrochloride and tropicamide, and the cornea was intermittently irrigated with balanced salt solution to prevent dehydration and allow electrical contact with the recording electrode. Rats were placed facing the stimulus at a distance of 20 cm. Recordings were completed within 20 min and animals were kept warm during and after the procedure. A reference electrode was placed through the ear, a grounding electrode was attached to the tail, and a gold electrode was placed in contact with the central cornea. A 15W red light was used to enable accurate electrode placement. This maneuver did not significantly affect dark adaptation and was switched off during the electrophysiological recordings. Scotopic ERGs were recorded from both eyes simultaneously and 10 responses to flashes of unattenuated white light (5 ms, 0.2 Hz) from a full-field/Ganzfeld stimulator (light-emitting diodes) set at maximum brightness (9 cd s/m^2^ without filter) were amplified, filtered (1.5-Hz low-pass filter, 1000 high-pass filter, notch activated) and averaged (Akonic BIO-PC, Buenos Aires, Argentina). The a-wave amplitude was measured as the difference in amplitude between the recording at onset and the trough of the negative deflection, and the b-wave amplitude was measured from the a-wave trough to the peak of the b-wave. Latency was measured from stimulus onset to the a-wave trough and b-wave peak. Runs were repeated 3 times with 5 min-intervals to confirm consistency. Mean peak latencies and peak-to-peak amplitudes of the responses from each group of rats were compared. Oscillatory potentials (OPs) were assessed as previously described [20]. Briefly, the same photic stimulator with a 0.2 Hz frequency and filters of high (300 Hz) or low (100 Hz) frequency were used. The amplitudes of the OPs were estimated by measuring the heights from the baseline drawn between the troughs of successive wavelets to their peaks. The sum of three OPs was used for statistical analysis.

### Histological Evaluation

Rats were anesthetized and intracardially perfused with saline solution, followed by a fixative solution containing 4% formaldehyde in 0.1 M PBS, pH 7.4. Then, the eyeballs were carefully removed and immersed for 24 hours in the same fixative. After dehydration, eyes were embedded in paraffin wax and sectioned (5 µm) along the vertical meridian through the optic nerve head. Microscopic images were digitally captured with a microscope (Eclipse E400, Nikon, Tokyo, Japan); 6-V halogen lamp, 20 W, equipped with a stabilized light source) and a camera (Coolpix s10; Nikon; Abingdon, VA, USA). Sections were stained with hematoxylin and eosin and were analyzed by masked observers. The average thickness (in µm) of the total retina was measured for each eye. Measurements were obtained at 1 mm dorsal and ventral from the optic disc, as previously described [18], [20]. For each eye, results obtained from four separate sections were averaged, and the mean of 5 eyes was recorded as the representative value for each group.

### Immunohistochemical studies

Antigen retrieval was performed by heating (90°C) slices for 30 minutes in citrate buffer (pH 6.3) and then preincubated with 2% normal horse serum, 0.1% BSA, and 0.4% Triton X-100 in 0.01 M PBS for 1 h. For specific immunodetection of retinal ganglion cells (RGC), paraffin sections were incubated overnight at 4°C with a goat policlonal anti-Brn3a antibody (1∶500; Santa Cruz Biotechnology, Buenos Aires, Argentina), and then, an anti-goat secondary antibody conjugated to Alexa Fluor 568 (1∶500; Molecular Probes, OR, USA) was used. After immunostaining, nuclei were stained with DAPI, as previously described [18].

For immunodetection of synaptophysin, paraffin sections were incubated overnight at 4°C with a rabbit polyclonal anti-synaptophysin (1∶500, Abcam, MA, USA), and then, a goat secondary antibody conjugated with Alexa Fluor 568 (1∶500, Molecular Probes, OR, USA) was used. For immunodetection of GFAP in Müller cells, paraffin sections were incubated overnight at 4°C with a mouse monoclonal anti-glial fibrillary acidic protein (GFAP) antibody conjugated to Cy3 (1∶1200; Sigma Chemical Co., St. Louis, MO, USA). For immunodetection of astrocytes, retinas were carefully detached and flat-mounted with the vitreous side up in superfrost microscope slides (Erie Scientific Company, New Hampshire, USA). Wholemount retinas were incubated overnight at 4°C with a mouse monoclonal anti-GFAP antibody, as previously stated. Specimens were mounted with antifade medium (Vectashield, Vector Laboratories, CA, USA) and viewed with a fluorescence microscope (BX-50, Olympus, Tokyo, Japan) mounted with a video camera (3CCD; Sony, Tokyo, Japan) attached to a computer running image analysis software (Optimus, Media Cybernetics, Silver Spring, MD, USA). Comparative digital images from different samples were grabbed using identical time exposition, brightness, and contrast settings. The area occupied by GFAP(+)-astrocytes was measured in a square areas corresponding to 0.01 mm^2^ and expressed as percentage of the total area. Results obtained from eight separate quadrants (four from the center and four from the periphery) were averaged, and the mean of 4 eyes was recorded as the representative value, as previously described [18], [20].

For vascular endothelial growth factor (VEGF) immunodetection, paraffin sections were treated with 0.3% H_2_O_2_ in PBS for 20 min (for blocking endogenous peroxidase activity) and incubated overnight at 4°C with a rabbit polyclonal anti-VEGF antibody (1∶800; Calbiochem, La Jolla, CA, USA). An immunohistochemical detection was performed using the LSAB2 System-HRP (Dako, California, USA), based on biotin-streptavidin-peroxidase, and visualized using 3,3′-diaminobenzidine (DAB) as chromogen, as described [20]. For immunodetection of BDNF, paraffin sections were incubated overnight at 4°C with a rabbit policlonal anti-BDNF antibody (1∶50; Santa Cruz Biotechnology, Buenos Aires, Argentina), and then, an anti-rabbit secondary antibody conjugated to Alexa Fluor 568 (1∶500; Molecular Probes, OR, USA) was used. BDNF(+) area was measured in transversal sections (area corresponding to 0.04 mm^2^) and expressed as percentage of the total area. Results obtained from four sections were averaged, and the mean of 5 eyes was recorded as the representative value. In all cases, some sections were treated without the primary antibodies to confirm specificity.

### Morphometric Analysis

All the images obtained were assembled and processed using Adobe Photoshop SC (Adobe Systems, San Jose, CA) to adjust the brightness and contrast. No other adjustments were made. For all morphometric image processing and analysis, digitalized captured TIFF images were transferred to ImageJ software version 1.42q (NIH, Bethesda, MD).

### Vascular Permeability

Vascular permeability was analyzed by measuring albumin-Evan's Blue complex leakage from retinal vessels as previously described [20]. Briefly, animals were anesthetized, and intracardiacally injected with a solution of Evan's Blue (2% w/v dissolved in PBS). Immediately after injection, animals turned visibly blue, confirming the dye uptake and distribution. After 40 min, animals were killed and flat-mounted retinas were obtained as described above. Microphotographs were obtained using identical time exposition, brightness, and contrast settings. For each group, results were qualitatively analyzed by comparing 5 eyes for group. Quantification of Evan's blue was assessed as previously described [21]. Briefly, 40 min after Evan's blue injection, the retina from one eye of each animal was removed, weighted (wet weight), and homogenized (1∶5 w/v) in phosphate saline buffer (PBS). The same volume of trichloroacetic acid (80%) was added to the homogenate which was centrifuged for 10 min at 9000 g (4°C). The supernatant was diluted with ethanol (1∶4) and its fluorescence was determined (excitation at 620 nm and emission at 680 nm). Calculations were based on external standards in the same solvent (10 to 200 ng/mL) and expressed as ng per milligram of tissue. For each group, the mean of 5 retinas were averaged, and recorded as the representative value.

### Western blotting

Retinas (one per condition) were homogenized in 100 µl of a buffer containing 10 mM HEPES, 1 mM EDTA, 1 mM EGTA, 10 mMKCl, Triton 0.5% (v/v), pH 7.9, supplemented with a cocktail of protease inhibitors (Sigma Chemical Co. St. Louis, MO, USA). After 15 min at 4°C, homogenates were gently vortexed for 15 seconds and centrifuged at 900 g for 10 min. Proteins (100 µg/sample) were separated in SDS, 12% polyacrylamide gel. After electrophoresis, proteins were transferred to polyvinylidenedifluoride membranes for 60 min at 15 V in a Bio-Rad Trans-Blot SD system (Bio-Rad Laboratories, Hercules, CA, USA). Membranes were blocked in 5% non-fat dry milk in Tris-buffered saline (pH 7.4), containing 0.1% Tween-20 for 60 min at room temperature and then incubated overnight at 4°C with a rabbit anti-VEGF antibody (1∶1000). Membranes were washed and then incubated for 1 h with a horseradish peroxidase-conjugated secondary antibody (1∶2000). Immunoblots were visualized by enhanced chemiluminescence Western blotting detection reagents (Amersham Biosciences, Buenos Aires, Argentina). Autoradiographical signals were quantified by densitometry using ImageQuant software and adjusted by the density of β-actin.

### TNFα levels assessment

Two retinas were homogenized in 150 µl of PBS, pH 7.0 supplemented with 10% fetal bovine serum heat inactivated and a cocktail of protease inhibitors. Samples were cleared by centrifugation for 10 min at 13,000 rpm. TNFα levels were determined as previously described [22], using specific rat enzyme-linked immunosorbent assays (ELISA) using antibodies and standards obtained from BD Biosciences, Pharmingen, San Diego, CA, USA, according to the manufacturer's instructions. The reaction was stopped and absorbance was read immediately at 450 nm on a microplate reader (Model 3550, BIO-RAD Laboratories, CA, USA).

### Measurement of thiobarbituric acid reactive substances (TBARS) levels

Retinas were homogenized in potassium buffer plus 60 mM KCl, pH 7.2 and TBARS levels were analyzed as previously described [22]. The reaction mixture contained: retinal homogenate, 10% SDS, and 0.8% thiobarbituric acid dissolved in 10% acetic acid (pH 3.5), and this solution was heated to 100°C for 60 min. After cooling, the precipitate was removed by centrifugation at 3200 g for 10 min. Then, 1.0 ml water and 5.0 ml of n-butanol-pyridine mixture (15∶1, vol/vol) were added, and the mixture was vigorously shaken and centrifuged at 2000 g for 15 min. The absorbance of the organic layer was measured at an emission wavelength of 553 nm by using an excitation wavelength of 515 nm with a Jasco FP 770 fluorescence spectrophotometer (Japan Spectroscopic Co. Ltd., Tokyo, Japan). The range of the standard curves of malondialdehydebis-dimethyl acetal (MDA) was 10–2000 pmol.

### Protein level assessment

Protein content was determined by the method of Lowry et al. [23], using BSA as the standard.

### Statistical analysis

The results were analyzed by two-way ANOVA in a completely randomized design (diabetes and EE). Comparisons were made with the Tukey's test. Results were considered significant at p<0.05.

## Results

The average body weight and blood glucose levels from animals injected with vehicle or STZ are shown in Table 1. A significant weight loss and an increase in blood glucose levels were observed at 3, 7, and 10 weeks after STZ injection, as compared with vehicle-injected rats. EE housing did not change these parameters in control (data not shown) or diabetic animals. At 10 weeks of diabetes, the occurrence of diabetic cataract was similar in SE- and EE-housed rats (Table 1).

**Table 1:** Average body weight, blood glucose concentration, and cataract prevalence in SE- or EE-housing animals.

Average of body weight (g)				Average of blood glucose concentration (mg/dl)		
Time After vehicle or STZ injection	Control	Diabetes+SE	Diabetes+EE	Control	Diabetes+SE	Diabetes+EE
0 days	352±6	355±9	358±9	113±8	115±11	119±10
3 weeks	411±14	357±13^a^	353±13^a^	112±4	508±22^b^	504±16^b^
7 weeks	481±17	369±12^b^	374±9^b^	118±4	585±10^b^	592±7^b^
10 weeks	517±11	371±19^b^	388±22^b^	114±3	566±20^b^	584±16^b^

Cataract prevalence (%)			
Time After vehicle or STZ injection	Control	Diabetes+SE	Diabetes+EE
0 days	0%	0%	0%
3 weeks	0%	0%	0%
7 weeks	0%	20%	20%
10 weeks	0%	70%	70%

Body weight and blood glucose levels in vehicle- or STZ-injected animals at different time points. STZ induced a significant decrease in body weight and an increase in blood glucose levels, which did not differ between SE- and EE-housed animals at all time points examined. Cataract occurrence was similar in both diabetic groups. Data are mean ± SEM (n = 10 animals per group).

a : p<0.05,

b : p<0.01 vs. aged-matched control animals, by Tukey's test.

**Figure 2:**
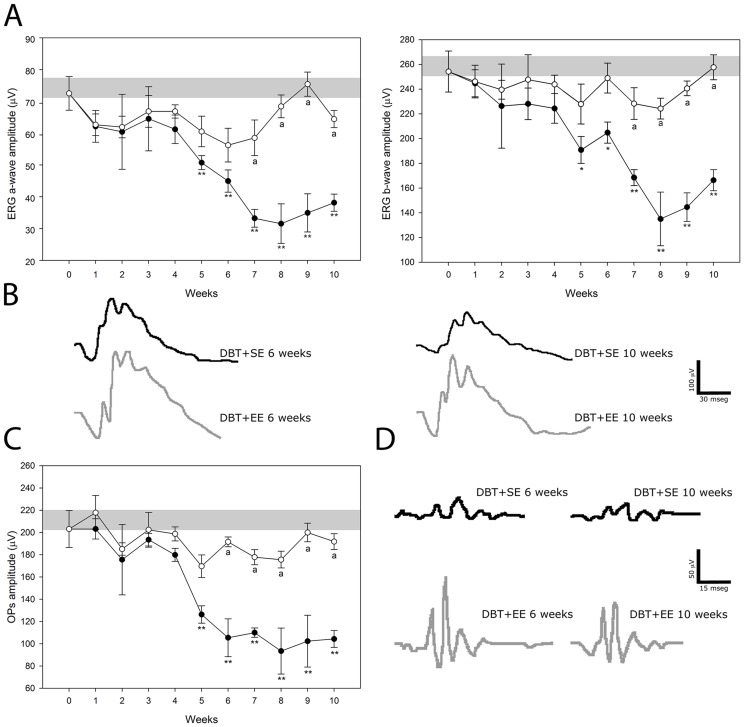
Effect of EE housing on retinal dysfunction induced by experimental diabetes. ERGs (A) and OPs (C) were weekly registered after the induction of diabetes. Experimental diabetes induced a significant decrease in ERG a- and b-wave amplitude in eyes from animals housed in SE (black circles), as compared with age-matched control animals (grey bar), after 5 weeks of STZ injection. In diabetic eyes from animals housed in EE (white circles), a significant prevention of these alterations was observed. Panel B shows representative scotopic ERG traces from control and diabetic animals housed in SE (DBT+SE) or EE (DBT+EE) assessed at 6 and 10 weeks after diabetes induction. A significant decrease in the sum of OP amplitude (C) was observed after 5 weeks of diabetes onset in eyes from animals housed in SE, while EE housing prevented the effect of diabetes on this parameter. Panel D shows representative OP traces from control and diabetic animals housed in SE or EE and assessed at 6 and 10 weeks after induction of diabetes. Data are the mean ± SEM (n = 10–12 animals per group); *p<0.05, **p<0.01 versus age-matched control; a: p<0.01 versus diabetic animals in SE, by Tukey's test.

Figure 2 depicts the electroretinographic activity in control and diabetic animals housed in SE or EE. In eyes from diabetic animals housed in SE, a gradual decrease in the scotopic ERG a-wave and b-wave amplitude was observed, which reached significance at 5 weeks post-injection of STZ. EE housing significantly prevented the diabetes-induced decrease in ERG a- and b-wave amplitude (Figure 2A). The ERG a- and b-wave latency did not differ among groups (data not shown). Representative ERG waveforms registered at 6 and 10 weeks of diabetes are shown in Figure 2B. The sum of OP amplitudes significantly decreased after 5 weeks of diabetes in animals housed in SE, as compared with vehicle-injected animals, whereas EE housing prevented the effect of diabetes on this parameter (Figure 2C and 2D). No significant differences in the ERG were observed between eyes from vehicle-injected animals housed in SE or in EE along the study (data not shown).

**Figure 3:**
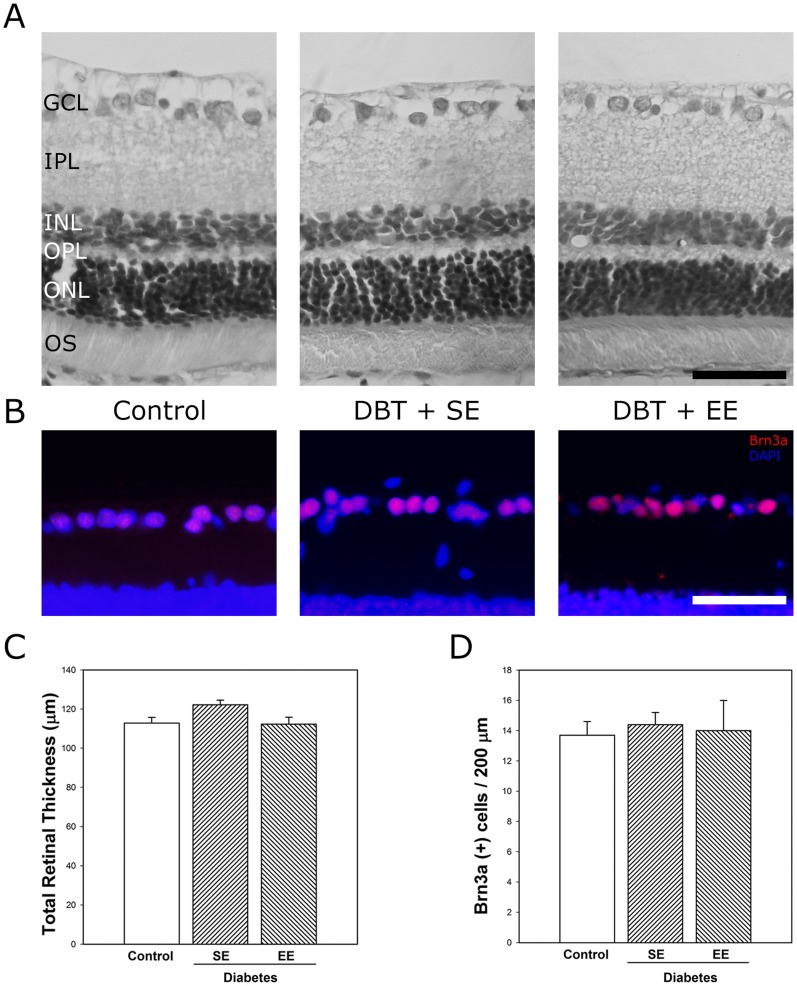
Retinal histology examination after 10 weeks of diabetes. Panel A: Representative photomicrographs of retinal sections from control eyes, and eyes from diabetic animals housed in SE (DBT+SE) or EE (DBT+EE). No evident alterations in retinal morphology were observed. Panel B: Immunohistochemical detection of Brn3a(+) cells in the GCL from a control eye, a diabetic eye from an animal housed in SE or EE. A strong Brn3a-immunostaining was confined to the GCL (red). Cell nuclei were counterstained with DAPI (blue). Panel C: Total retinal thickness and RGC count evaluated by Brn3a immunostaining. These parameters did not differ among retinas from control animals housed in SE (control) and retinas from diabetic animals housed in SE or EE. Scale bar: 50 µm. Data are the mean ± SEM (n = 5 eyes per group). GCL, ganglion cell layer; IPL, inner plexiform layer; INL, inner nuclear layer; ONL, outer nuclear layer; OS, outer segments of photoreceptors.

**Figure 4:**
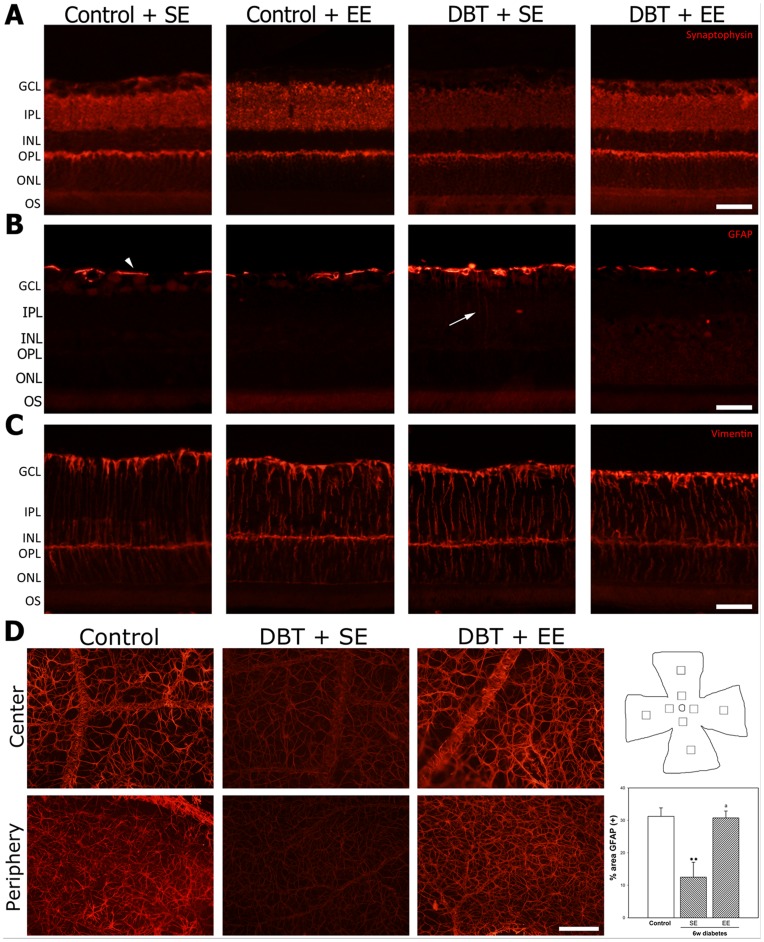
Effect of EE housing on retinal synaptophysin- and Müller cell and astrocyte GFAP-immunoreactivity. Panel A: Representative photomicrographs of retinal sections from a vehicle injected animal housed in SE or EE, and a diabetic animal housed in SE or EE. At 6 weeks of experimental diabetes, a decrease in IPL synaptophysin levels was observed in SE-housed animals, whereas EE housing prevented the decrease in synaptophysin immunoreactivity in the IPL from diabetic rats. No evident differences in synaptophysin levels were observed in the ONL among groups. In non-diabetic animals, EE did not affect synaptophysin immunoreactivity. Shown are photographs representative of four eyes per group. Panel B: In SE-housed diabetic animals, a slight increase in Müller cell process GFAP levels (arrow) was observed which was not evident in EE-housed diabetic animals Panel C: no changes in vimentin-immunoreactivity were observed among groups. Panel D: Assessment of astrocyte GFAP-immunoreactivity in flat-mounted retinas after 6 weeks of diabetes induction. In control retinas, astrocytes were intensely immunoreactive for GFAP in the central and peripheral regions. The intensity of GFAP-immunoreactivity was significantly reduced in eyes from diabetic animals housed in SE, both in the peripapillary and the peripheral regions. In eyes from diabetic animals housed in EE, a prevention of the decrease in GFAP-immunoreactivity induced by diabetes was observed both in central and peripheral regions. Right panel: Schematic diagram of a flat-mounted retina showing all the regions analyzed. Assessment of the GFAP immunoreactivity area. Data are the mean ± SEM (n = 5 eyes per group); **p<0.01, versus age-matched controls; a: p<0.01, versus diabetic animals housed in SE, by Tukey's test. Scale bar: 50 µm for panel A, B and C, and scale bar: 100 µm for panel D.

At 10 weeks after vehicle or STZ injection, retinal thickness (Figure 3A and 3C), and the number of Brn3a(+) ganglion cell layer (GCL) cells (Figure 3B and 3D) did not differ among groups. EE housing of non-diabetic rats did not affect these parameters (data not shown). In order to analyze the effect of experimental diabetes and EE on retinal synapses, synaptophysin immunoreactivity was analyzed in retinas from non-diabetic and diabetic animals housed in SE or EE. Intense immunostaining for synaptophysin was observed in both plexiform layers from non-diabetic animals housed in SE or EE (Figure 4A), whereas in retinas from SE-housed animals that were diabetic for 6 weeks, a weak immunolabeling was observed in the inner plexiform layer (IPL). EE housing prevented the effect of experimental diabetes on synaptophysin immunoreactivity in the IPL. Retinal immunoreactivity for GFAP and vimentin were analyzed at 6 weeks of diabetes in animals housed in SE or EE. In SE-housed animals, experimental diabetes induced a slight increase in retinal GFAP- immunoreactivity associated with activated Müller cells (Figure 4B), whereas EE housing prevented the increase in this parameter. Vimentin-immunostaining did not change among experimental groups (Figure 4C). In flat-mounted retinas from non-diabetic animals, an intense GFAP-immunoreactivity was observed in star-shaped astrocytes forming a single cell layer adjacent to the inner limiting membrane (Figure 4D). At 6 weeks of diabetes, GFAP-immunoreactivity in central and peripheral retina clearly decreased in SE-housed animals, whereas in retinas from EE-housed diabetic animals, GFAP-immunoreactivity was similar to that observed in non-diabetic eyes, as shown in Figure 4D. EE housing did not modify astrocyte GFAP levels in non-diabetic animals (data not shown).

**Figure 5:**
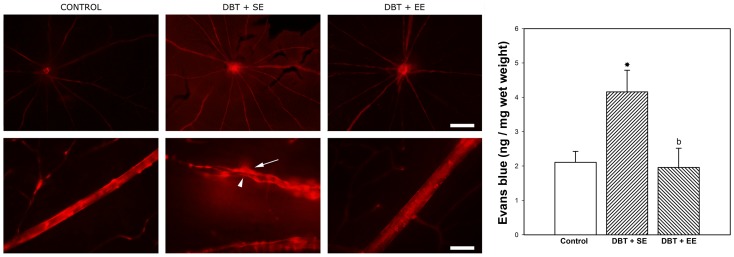
Effect of EE on BRB integrity. Left panel: Visualization of retinal blood vessel leakage after intravascular perfusion with Evan's blue. In control retinas, the large vessels were sharply outlined without the presence of the dye outside the lumen. At 6 weeks of diabetes, in retinas from animals housed in SE (DBT+SE), focal sites of leakage were noticed (arrow), with the dye diffusely distributed through the retinal parenchyma. Focal varicosities (arrowhead) were also noticed. In eyes from diabetic animals housed in EE (DBT+EE), vascular permeability and large vessel morphology were similar to control (non-diabetic) animals. Shown are microphotographs 40× (upper panel A) and 400× (lower panel A), representative of 5 eyes/group. Scale bar: 500 µm (upper panel) and 50 µm (lower panel). Right panel: Assessment of retinal Evan's blue levels. In SE-housed animals, diabetes induced a significant increase in this parameter, which was significantly reduced in diabetic animals housed in EE. Data are the mean ± SEM (n = 5 eyes per group); *p<0.05, versus age-matched controls; b: p<0.05, versus diabetic animals housed in SE, by Tukey's test.

The Evan's blue-albumin complex leakage method was used to assess the blood retinal barrier (BRB) integrity in flat-mounted retinas at 6 weeks after STZ injection (Figure 5). In non-diabetic animals, the dye was exclusively localized within the vessel lumen of the retinal vascular network, with very low to complete absence of background fluorescence level. Large vessels showed straight walls without leakage of the dye. In diabetic animals housed in SE, a generalized leakiness (red background) as well as focal dye leakage from the optic disk region and larger vessels was observed. Moreover, constrictions and varicosities, characteristic of diabetes, were observed in large vessels. In eyes from EE-housed diabetic animals, there was no evidence of BRB integrity loss, and large vessels morphology was preserved. In diabetic animals housed in SE, retinal levels of Evan's blue significantly increased as compared with non-diabetic animals, whereas in diabetic animals housed in EE, retinal Evan's blue levels were similar to those found in control (non-diabetic) animals, as shown in the right panel of Figure 5. EE housing in non-diabetic animals did not change retinal Evan's blue levels (data not shown).

**Figure 6:**
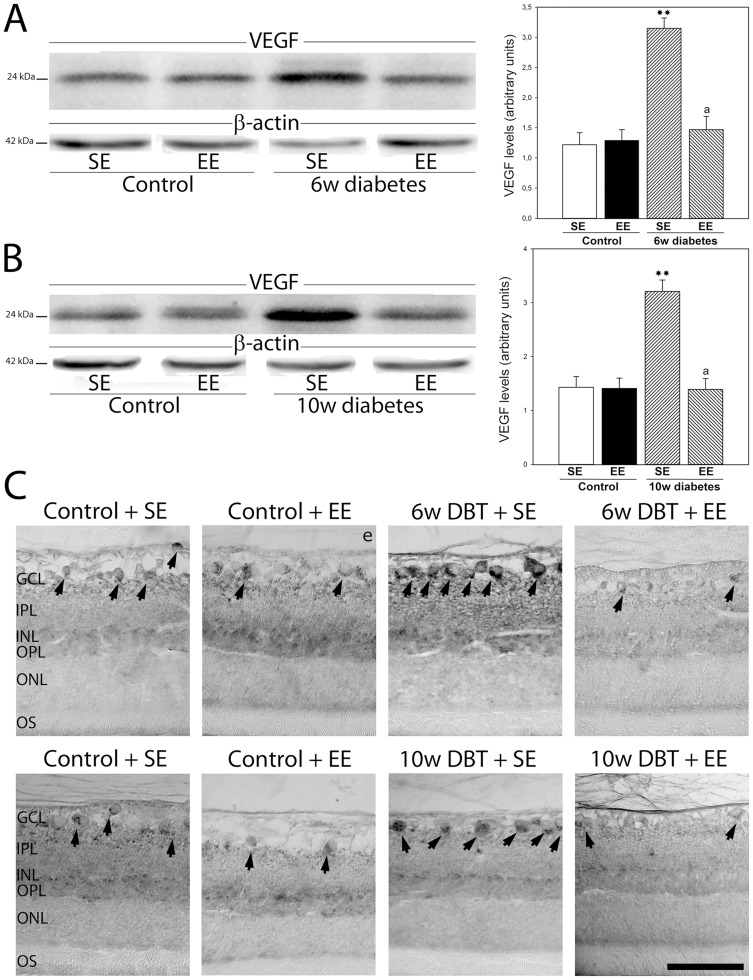
Effect of EE housing on retinal VEGF levels. Panel A: representative Western blot performed at 6 weeks after the injection of STZ. VEGF levels significantly increased in retinas from diabetic animals housed in SE as compared with age-matched controls. The increase in VEGF levels induced by diabetes was totally prevented by EE housing. A similar profile was observed in animals that were diabetic for 10 weeks (Panel B). Densitometric analysis for both time points is shown on the right. Data are the mean ± SEM (n = 5 animals per group); **p<0.01 versus age-matched controls; a: p<0.01 versus diabetic animals housed in SE, by Tukey's test. Panel C: Localization of VEGF in retinal sections, at 6 and 10 weeks after the injection of vehicle or STZ. In control retinas, a weak immunoreactivity was diffusely observed throughout the inner retina and in the GCL (arrow). In retinas from diabetic rats housed in SE, an intense immunoreactivity in the inner plexiform layer, some cells in the inner nuclear layer and perikarya in GCL cells (arrows) was observed both 6 and 10 weeks after diabetes induction. No immunoreactivity for VEGF was observed in the outer retina. EE housing prevented the effect of diabetes on VEGF-immunoreactivity. Shown are photographs representative of four eyes per group.

Retinal VEGF protein levels and localization were analyzed by Western blot and immunohistochemistry, respectively (Figure 6). In SE-housed animals that were diabetic for 6 weeks, VEGF levels significantly increased in comparison with control retinas. EE housing significantly prevented the effect of diabetes on VEGF, reaching similar levels to those observed in control animals (Figure 6A). A similar profile was observed after 10 weeks of diabetes (Figure 5B). Immunohistochemical studies confirmed these results (Figure 6C). In control animals, a weak VEGF-immunoreactivity was observed in GCL and IPL. After 6 weeks of diabetes, an intense immunolabeling was observed in GCL cells, IPL, and some cells in the inner nuclear layer (INL). In EE-housed diabetic animals, VEGF-immunoreactivity was similar to that observed in non-diabetic eyes. A similar profile was found in retinas from STZ-injected rats at 10 weeks post-injection. There were no differences in VEGF protein levels and immunoreactivity between vehicle-injected animals housed in SE or EE at both time points.

**Figure 7:**
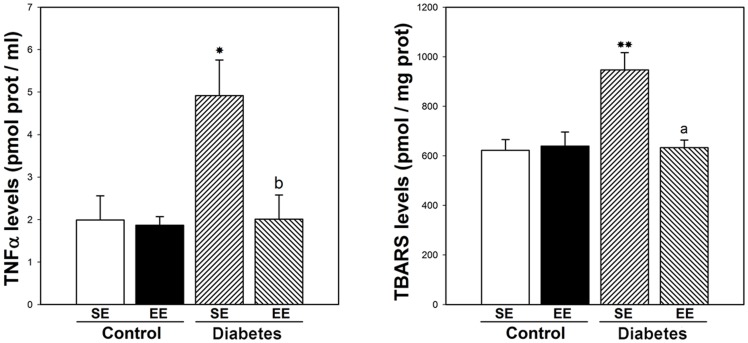
Effect of EE housing on retinal TNFα and TBARS levels. In SE-housed animals, diabetes induced a significant increase in TNFα and lipid peroxidation levels, whereas EE housing totally prevented the increase in these parameters. Data are mean ± SEM (n = 5 animals per group), *p<0.05 versus age-matched controls; b: p<0.05, a: p<0.01 vs. diabetic animals housed in SE, by Tukey's test.

TNFα levels were evaluated at 6 weeks after STZ injection. In retinas from SE-housed animals, experimental diabetes induced a significant increase in TNFα levels, whereas EE housing prevented the effect of diabetes on this parameter, as shown in Figure 7. Retinal lipid peroxidation was assessed at 6 weeks after STZ injection. In SE, experimental diabetes induced a significant increase in TBARS levels, which was prevented by EE housing (Figure 7).

**Figure 8:**
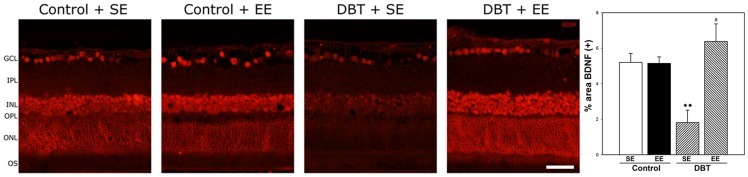
Expression of BDNF in retinas after 6 weeks of diabetes onset in SE- or EE-housed animals. In animals housed in SE a decrease in BDNF immunostaining in both inner and outer retina was observed, whereas in retinas from diabetic animals housed in EE, BDNF expression was similar to control animals kept in SE or EE. Shown are photographs representative of four eyes per group. Right panel: Assessment of the BDNF(+) immunoreactivity area. In SE-housed animals, experimental diabetes induced a decrease in BDNF(+) area which was prevented in diabetic animals housed in EE. Data are the mean ± SEM (n = 5 eyes per group); **p<0.01, versus age-matched controls; a: p<0.01, versus diabetic animals housed in SE, by Tukey's test. Scale bar: 50 µm. GCL, ganglion cell layer; IPL, inner plexiform layer; INL, inner nuclear layer; ONL, outer nuclear layer; OS, outer segments of photoreceptors.

**Figure 9:**
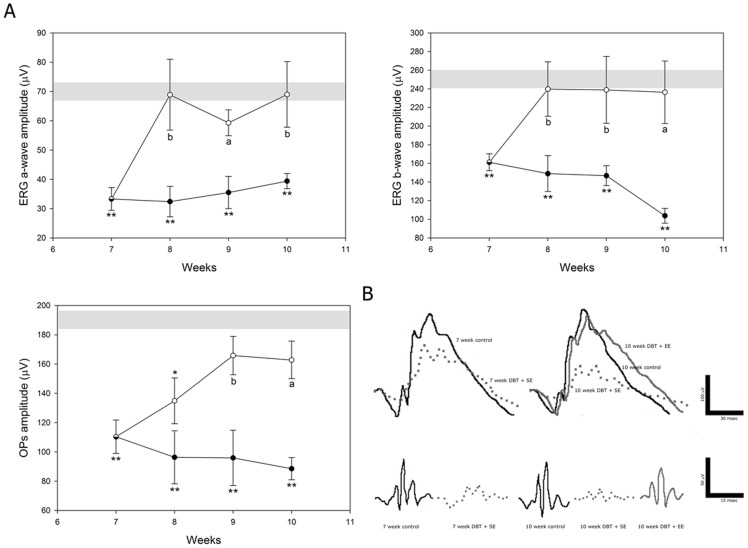
Effect of EE housing started at 7 weeks after diabetes onset on retinal function. In diabetic animals kept in SE (black circles), a significant decrease in scotopic ERG a- and b-wave amplitude which progressed over time until 10 weeks as compared with non-diabetic animals (grey bar) was observed, whereas a significant protection of retinal function was already evident after 1 week of EE housing (white circles). For OP amplitude, EE housing reversed the decrease induced by STZ at 2 weeks after EE exposure, and the protection induced by EE housing persisted until week 10. Data are the mean ± SEM (n = 10–12 animals per group); *p<0.05, **p<0.01 versus non diabetic eyes; a: p<0.01, b: p<0.05 versus diabetic eyes housed in SE, by Tukey's test. Panel B shows representative scotopic ERG and OP traces.

Retinal BDNF immunoreactivity was evaluated at 6 weeks after STZ injection. No differences in BDNF immunoreactivity were found between control retinas from animals housed in SE or EE (Figure 8). In SE-housed animals, diabetes induced a significant decrease in BDNF immunoreactivity, affecting the whole retinal thickness, whereas in EE-housed diabetic animals, BDNF immunoreactivity was similar to that observed in non-diabetic animals housed in SE or EE, as shown in Figure 8. The therapeutic effect of environmental enrichment was analyzed by housing animals with 7 weeks of diabetes in EE. EE provoked a significant reversion of the diabetes-induced decrease in the ERG a- wave, b-wave, and OP amplitude, as shown in Figure 9.

## Discussion

DR is a major cause of visual loss. In this study, instead of a pharmacological or surgical approach, we housed adult diabetic animals in EE. Notably, environmental enrichment did not affect the weight loss and hyperglycemia, as well as the occurrence of cataracts induced by experimental diabetes. However, the functional and histological measurements carried out to test the effects of EE on early retinal damage induced by diabetes, consistently showed clear improvements with respect to SE-housed diabetic animal: ERG recordings confirmed preservation of retinal function, and structural studies showed protection of inner retina synapses, and BRB integrity.

ERG is the most frequently used noninvasive measurement for the assessment of retinal function. Alterations in ERG responses have been shown in both animals and humans with diabetes [20], [24], [25]. EE housing which did not show any effect in control retinas, prevented the progressive decrease in ERG a-wave, b-wave, and OP amplitude induced by experimental diabetes.

Although it is becoming increasingly clear that retinal cells are affected by diabetes, the retinal cell degeneration rate varies in different experimental models, with a clear progression over the duration of diabetes. Barber et al. [26] demonstrated that the total number of cell bodies in the GCL is reduced by 10% after 7.5 months of diabetes. In our experimental conditions, at least at 10 weeks of experimental diabetes, no differences in retinal morphology and RGC number between diabetic and non-diabetic groups were observed. In this sense, one limitation to the conclusions that can be drawn from this study relies on the fact that the relatively short duration of diabetes (i.e. 10 weeks of diabetes) may be not sufficient to produce irreversible retinal changes. However, in our hands, *Wistar* rats with hyperglycemia in a range of ∼500 mg/dl for longer periods became considerably sick, which can affect their differential behavior in SE and EE cages, and therefore the beneficial effects of EE housing could be masked.

Synaptophysin, a highly abundant integral presynaptic vesicle membrane protein, is commonly used as a quantifiable proxy for functional presynaptic terminals [27], [28]. Despite the preservation of RCG number, a decrease in synaptophysin immunoreactivity was observed in the retinal IPL from diabetic animals housed in SE at early stages of diabetes (i.e., 6 weeks after STZ injection), in agreement with other reports [29], [30]. EE housing significantly prevented the decrease in synaptophysin immunoreactivity induced by diabetes, supporting a preservation of synaptic connections between inner retinal neurons.

Retinal glia has been implicated in early stages of DR (reviewed in [31]). In agreement with the results shown herein, it has been previously demonstrated that experimental diabetes increases GFAP levels in Müller cells and decreases its expression in astrocytes [32]. Müller cells participate in the establishment of the BRB, and a disturbance of this function is assumed to contribute to vessel leakage in the diabetic retina [31]. Müller cells that do not express GFAP under physiological conditions are known to express GFAP in pathological situations, including experimental type 1 diabetes [32], [33]. In SE-housed animals, experimental diabetes provoked a moderate gliosis in Müller cells, as shown by an increase in GFAP-immunoreactivity in these cells, which was prevented by EE housing. Astrocytes are in close association to vessels and capillaries, contributing to maintain their integrity, and increasing retinal vascular endothelium barrier properties. In agreement with previous reports [20], [32], experimental diabetes induced a decrease in astrocyte GFAP-immunoreactivity in SE- housed rats which was not observed in EE-housed diabetic animals.

Evan's blue binds irreversibly to serum albumin; thus, its distribution reflects albumin exchange between the intra- and extravascular compartments. Evan's blue has been widely used for BRB integrity studies [34], [35]. As previously described [20], in the retina from SE-housed diabetic animals, extravasated Evan's blue was observed, whereas EE-housing protected the BRB integrity. Since a reduction of GFAP-expression in retinal astrocytes and an increase in Müller cell GFAP levels during diabetes were linked to a reduced ability to maintain BRB integrity [31], [32], it seems likely that changes in GFAP-immunoreactivity in Müller cells and astrocytes could be involved in Evan's blue leakage in eyes from SE-housed diabetic rats, and for the preservation of BRB integrity induced by EE.

The present results suggest that at retinal level, EE may act through different pathways, particularly regulating retinal VEGF, TNFα, oxidative stress, and BDNF levels. Many lines of evidence support that VEGF, as an angiogenesis inducer, plays a key role in DR, and its role in the BRB breakdown is well established [36]. Intravitreal VEGF injections in monkey eyes reproduce many features of both non proliferative and proliferative DR [37]. Anti-VEGF therapies show promising benefits against advanced stages of DR [38], [39]. Since EE-housing prevented the increase in retinal VEGF levels induced by diabetes, it seems likely that VEGF could be a target for the protective effect of EE.

Both inflammation and oxidative stress play a significant role in the pathobiology of DR [40], [41]. In this sense, a significant increase in retinal TNFα levels and lipid peroxidation was observed in SE-housed diabetic animals, whereas EE prevented the increase in these parameters. In agreement, it was recently shown that EE housing attenuates the increase in hippocampal TNFα levels in a mouse model of influenza infection [42], and decreases lipid peroxidation after transient middle cerebral artery occlusion in rats [43].

BDNF plays a major role in EE-mediated neuroprotection. Landi et al. [44] have shown that an increase in BDNF expression is necessary for EE effects on the maturation of retinal acuity. In the adult, BDNF expression that enhances and supports brain plasticity is increased by EE housing [45]–[47]. It was shown that serum and retinal levels of BDNF are significantly reduced at early stages of diabetes in rats [48], and BDNF protects retinal neurons from hyperglycemia *in vitro* [49], supporting that decreased BDNF levels may damage retinal cells early in the course of diabetes. In agreement, BDNF-immunoreactivity decreased in the retina from SE-housed diabetic animals as compared with non-diabetic animals, whereas EE housing prevented the effect of experimental diabetes on BDNF immunoreactivity. In retina, there are two sources of endogenous BDNF: one source is the local production of BDNF (mainly by Müller cells), and the other source is BDNF retrogradely transported from the brain, that reaches RGC [50]. Since a pan-retinal increase in BDNF levels was observed in EE-housed diabetic animals, the present results suggest that both BDNF sources could be involved in retinal diabetic damage and in its prevention by EE housing. In this way, BDNF produced by Müller cells which span the entire thickness of the retina, could reach the inner and outer nuclear layers, while BDNF retrogradely transported from the brain could increase the neurotrophin levels in RGC from diabetic animals housed in EE. LaVailet al. [51] reported that photoreceptors could be significantly protected from the damaging effects of light by intravitreal injection of BDNF. This finding could suggest that the ERG preservation in diabetic animals housed in EE could involve a BDNF-dependent mechanism. In agreement, it was demonstrated that expression of BDNF resulted in a significant delay in photoreceptor cell death, and maintenance of retinal function assessed by ERG recording in a model of primary rod photoreceptor degeneration [52]. Monocular deprivation decreases BDNF levels in the deprived eye [53], which suggests that visual experience with its associated neuroelectric activity have an impact on BDNF expression in the retina. Since EE is visually complex in comparison with typical laboratory home cages, the increase in sensorial activity of rats kept in EE could provoke a higher production of the neurotrophin in diabetic retinas. Some possible factors that may increase BDNF expression would be illumination, contrast, spatial frequency and the widened area of the accessible environment that may increase the range of depth perception [52]. Thus, although the possibility that EE might impact other systemic effects of diabetes, such as insulin resistance, HbA1c, blood pressure, and stress hormone expression cannot be formally ruled out, the present results suggest that EE behaved as an anti-VEGF, anti-inflammatory, anti-oxidative therapy, and increased BDNF in the context of retinal damage induced by early diabetes. In addition, EE favors stress-free and challenge-free interactions in a stimulating surrounding that might enhance self-defense responses of the retina to disease [16]. Moreover, considering the higher size of EE cages, an increased physical activity could also contribute to the beneficial effects of EE housing.

DR is a complex disease which involves many pathological pathways; thus, therapeutic strategies with multiple modes of actions are likely more effective in the management of the disease. Taken together, these results indicate that a significant prevention of early retinal changes induced by diabetes can be achieved by EE housing. However, the fact that EE housing started before the appearance of retinal changes provoked by experimental diabetes could limit the relevance of these results. Therefore, in order to analyze whether environmental enrichment could not only prevent but also reduce DR progression, we housed diabetic animals at 7 weeks of diabetes onset, a time point at which functional alterations were already evident. As shown herein, a short term exposure to EE already induced a significant protection of the retinal function (ERG) in diabetic rats. These results indicate that the EE not only preserved, but also restored the retinal function, supporting that EE could be capable of suppressing actively ongoing retinal damage. It should be noted that at 10 weeks after STZ injection, some SE- and EE-housed diabetic animals developed cataract. However, since the incidence of cataract was similar in SE- and EE-housed rats, it seems unlikely that the occurrence of cataract could account for the protective effect of EE on retinal function at 10 weeks of diabetes.

Current data remain incapable of addressing which components of EE (motor, sensory, cognitive, or social stimulation) are responsible for the retinal protection against early diabetic damage, but ongoing studies by our group are in progress to analyze this issue. Despite that more studies are required to define the mechanism responsible for the retinal protection of adult diabetic animals in enriched conditions, the non-invasive nature of EE makes this tool particularly worthy of further examination.
